# Reduced TIGIT Expression on T Cells Links Hyperglycemia to Immune Dysregulation in Type 1 Diabetes

**DOI:** 10.3390/cells15020195

**Published:** 2026-01-20

**Authors:** Martyna Tomaszewicz, Anna Ronowska, Julia Strzelecka, Agnieszka Jankowska-Kulawy, Katarzyna Stefańska, Piotr Trzonkowski, Maciej Zieliński

**Affiliations:** 1 Department of Medical Immunology, Faculty of Medicine, Medical University of Gdańsk, Marii Skłodowskiej Curie 3a Street, 80-210 Gdańsk, Poland; 2 Department of Laboratory Medicine, Faculty of Medicine, Medical University of Gdańsk, Marii Skłodowskiej Curie 3a Street, 80-210 Gdańsk, Poland; 3 Poltreg Spółka Akcyjna, Botaniczna 20 Street, 80-298 Gdańsk, Poland; 4 Department of Obstetrics and Gynecology, Faculty of Medicine, Medical University of Gdańsk, Marii Skłodowskiej Curie 3a Street, 80-210 Gdańsk, Poland

**Keywords:** T regulatory cells, type 1 diabetes, hyperglycemia, TIGIT, immunometabolism

## Abstract

**Highlights:**

**What are the main findings?**

- Type 1 diabetes is characterized by a lower percentage of TIGIT^+^ CD4-positive T cells.
- Hyperglycemia reduces the frequency of TIGIT+ and CTLA-4+ T regulatory cells.

**What are the implications of the main findings?**

- Reduced TIGIT+ Tregs in type 1 diabetes is associated with impaired glucose metabolism.
- Hyperglycemia may weaken immune tolerance by targeting TIGIT+ Tregs.
- Loss of TIGIT+ Tconvs under high glucose may promote autoimmune activity.

**Abstract:**

T cells play an important role in the development and progression of type 1 diabetes (T1D). Checkpoint receptors regulate T cell activity, and their expression may be linked to the cells’ metabolic state. This study aims to investigate the association between T regulatory (Treg) and T conventional (Tconv) cells expressing various checkpoint inhibitors and glucose metabolism in type 1 diabetes patients and healthy controls (HCs). The study included 28 participants, with 16 of them diagnosed with type 1 diabetes, while 12 constituted a healthy control group. Multicolor flow cytometry, spectrophotometric analysis, and bead-based multiplex assays were utilized for the analyses. The study revealed that the most significant difference in T cell subsets in peripheral blood concerned TIGIT. Compared to healthy subjects, the percentages of TIGIT+ Tregs and TIGIT+ Tconvs were lower in T1D patients. Interestingly, hyperglycemia in in vitro cultures reduced percentages of TIGIT+ Tregs and TIGIT+ Tconvs, and to some extent also CTLA-4+ Tregs. A decreased percentage of these subsets was, in turn, associated with reduced glucose uptake and lower activity of the enzymes responsible for various stages of glucose metabolism. The described associations suggest a negative influence of hyperglycemia in T1D on immune regulation via a TIGIT-dependent mechanism. Hyperglycemia seems to reduce the percentage of highly regulatory TIGIT+ Tregs both in vivo and in vitro, and it is associated with reduced glucose consumption by these cells. At the same time, a reduction in the percentage of TIGIT+ Tconvs under such conditions may facilitate higher activity of Tconvs, including aberrant autoimmune reactions.

## 1. Introduction

Basically, type 1 diabetes [T1D] is an autoimmune disorder with roots in T cell-mediated destruction of β cells in the pancreas. As scientific understanding advances, it is clear that T1D is not simply an immune dysfunction but a complex interplay of various factors, including genetics, the immune system, the microbiome, and environmental influences, with variation among individuals [1]. While the health and quality of life of T1D patients have improved in recent decades, there is still a strong need to develop new treatment methods [1]. One alternative treatment option is immunotherapy using in vitro expanded autologous polyclonal or antigen-specific T regulatory cells [2,3]. These cells suppress an overactive immune response against the body’s own tissues and promote immune tolerance [4].

T regulatory cells are characterized by high expression of CD25 (IL-2 receptor) and the transcription factor FoxP3. Their immunosuppressive function is exerted through the production of suppressive cytokines (IL-10, TGF-β, and IL-35) and granzymes, or direct cell-to-cell contact involving TCR, costimulatory receptors (e.g., CD28), and immune checkpoint inhibitors (e.g., cytotoxic T lymphocyte antigen 4, CTLA-4; programmed cell death receptor 1, PD-1; and T cell immunoreceptor with Ig and ITIM domains, TIGIT) [5,6]. The interaction of immune checkpoints with their ligands plays a key role in shutting down conventional T cells (effector lymphocytes), the main players in T cell-mediated inflammation, thus maintaining tissue homeostasis and guarding against a shift towards autoimmunity.

The role of T cell immunoglobulin and ITIM domain (TIGIT) in autoimmunity is increasingly recognized as a significant area of research, highlighting its potential as a therapeutic target. TIGIT functions as an immune checkpoint that modulates T cell activity, promoting self-tolerance and preventing autoimmune reactions.

Multiple meta-analyses confirm the relationship between immune checkpoint gene polymorphisms and an increased risk of autoimmune disease occurrence [7]. There are indications that the expression of CD226, a competitor of TIGIT for CD155, is negatively correlated with Treg cell function and may predispose individuals to autoimmune diseases [8,9]. Additionally, agonistic antibodies against TIGIT have been shown to alleviate the severity of autoimmune diseases [6,10]. The relevance of immune checkpoints is not limited to autoimmune disease treatment options but may also serve as a prognostic biomarker for therapy outcomes [11], which has been proven in a study on T1D clinical trial patients using teplizumab [12].

In this study, our objective was to determine if there are any phenotypes or metabolic patterns in Treg cells characteristic of autoimmune diseases (such as type 1 diabetes) or health. Additionally, as the activation of T cells is regulated via checkpoint molecules, we aimed to find an association between their expression and different metabolomic patterns represented as signaling pathway protein phosphorylation or enzymatic activities. This approach aimed to identify the metabolic element that predominates in a given cell subpopulation, taking into account environmental conditions, as well as to determine the relationship between the expression of immune checkpoint receptors and the dominant metabolic pathway governing cell activity.

## 2. Materials and Methods

### 2.1. Study Design

In this study, 28 patients were enrolled between October 2020 and August 2023. Sixteen of them were diagnosed with type 1 diabetes, while twelve of them constituted a healthy control group (Table 1). T1D patients were recruited by the Department of Pediatric Diabetology and Endocrinology, Medical University of Gdańsk. The healthy control patients’ samples were obtained from the Regional Centre for Blood Donation and Treatment in Gdańsk. All study participants were of Caucasian ethnicity. All blood donors gave informed consent, and all experimental protocols were approved by the Independent Bioethics Commission for Research of the Medical University of Gdańsk (agreement no. NKBBN/414/2018). The study was conducted in accordance with the guidelines of the Commission and the Declaration of Helsinki.

### 2.2. Cell Isolation, Sorting, and Culture

The buffy coat from blood products from T1D patients and healthy volunteers was collected for the analysis. We used Ficoll Paque Plus (GE Healthcare, Chicago, IL, USA) density gradient centrifugation to separate cell fractions and obtain peripheral blood mononuclear cells (PBMCs). The isolated cells were washed in phosphate-buffered saline (PBS). Next, the EasySep™ Human CD4+ T Cell Negative Isolation Kit (StemCell, Vancouver, BC, Canada) was used to isolate CD4-positive cells from PBMCs following the manufacturer’s protocol. CD4+ cells were washed, suspended in a small amount of PBS, and stained using the following combination of antibodies: CD4 (RPA-T4), CD127 (HIL-7R-M21), and CD25 (2A3). The stained cells were sorted with the BD FACS Aria cell sorter (BD Bioscience, Milpitas, CA, USA) or the FACS Influx sorter (BD Bioscience, Franklin Lakes, NJ, USA) into Treg (CD4+CD127-CD25high, doublets-), and T conventional cells (CD4+CD127+CD25-, doublets-). After sorting, cell purity was checked with the BDLSR Fortessa Flow Cytometer (BD Bioscience, Milpitas, CA, USA), and the acceptance criterion was set to a minimum of 95% of the sorted population. Cells were suspended in an XVIVO20 (Lonza, Verviers, Belgium) or RPMI (Lonza, Verviers, Belgium) cell culture medium with 10% heat-inactivated serum (autologous in T1D patients and pooled serum in A/B/0 groups in the healthy control group), 9000 IU/mL of IL-2 (Proleukin, Novartis, Nürnberg, Germany), and were stimulated using anti-CD3 and anti-CD28 coated beads (MACS GMP ExpAct Treg Kit, Miltenyi Biotec, Bergisch Gladbach, Germany) at day 0 and day 5 in a 1:1 bead-to-cell ratio. Cells were cultured according to the previously published protocol [11,13,14]. Cells were cultured for 12 days to obtain adequate cell numbers to perform the planned analysis.

The study was divided into two stages. In the first one (stage 1), we cultured cells from T1D patients and healthy controls in the XVIVO 20 cell culture medium (Lonza, Verviers, Belgium). The second stage (stage 2) involved modifying the cell culture conditions to obtain strictly defined glucose concentrations of 0 mg/dL, 100 mg/dL, and 1000 mg/dL in RPMI1640 (Lonza, Verviers, Belgium) (*n* = 7). On day 12 cells were harvested, washed, and divided into three fractions:

- 1–2 mln cells suspended in PBS for phenotype analysis;
- 1–2 mln cells dry-pellet-frozen for mTOR and p38 MAPK phosphorylation analysis;
- 1–40 mln cells suspended in PBS (concentration: 10 mln cells in 200 uL of PBS) for enzymatic activity analysis.

### 2.3. Flow Cytometry

Cells were analyzed with the BD LSR Fortessa Flow Cytometer (BD Bioscience, Milpitas, CA, USA). We used minimum backbone markers for the gating strategy, namely, CD4 and CD3 (Supplementary Figure S6). The panel also included CD45RA, CD62L, CD27, CD279 (PD-1), CD178 (FASL), CD28, CD57, CD195 (CCR5), CD184 (CXCR4), CD39, CD152 (CTLA-4), TIGIT, FoxP3, Helios, GLUT 1, GLUT 3, MitoTracker Green, and MitoTracker Red CMXRos, according to Table 2.

**Figure 1:**
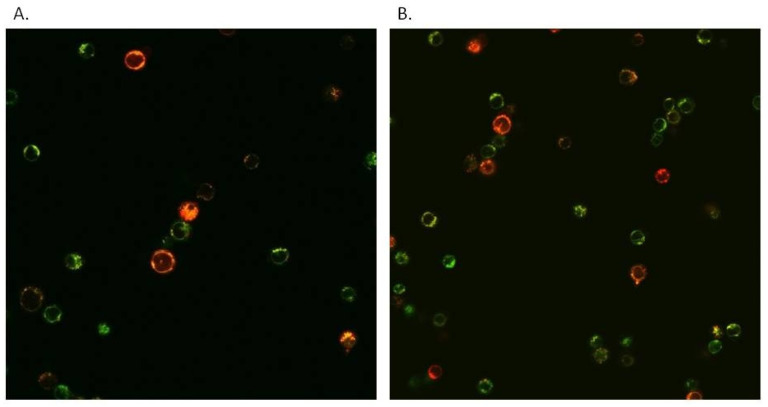
The visualization of Mitotracker Green (green) and Mitotracker Red CMXRos (red) staining (merged) of Treg (**A**) and Tconv cells (**B**).

Mitotracker Red and Mitotracker Green staining of Treg cells (merged, Figure 1A) and Tconv cells (merged, Figure 1B) was confirmed and visualized by the FLUOVIEW FV3000 confocal microscope (Olympus; Tokyo, Japan).

### 2.4. Spectrometry—Enzymatic Activity

Before the assay, samples were diluted to the desired protein concentration, which varied from 1 to 3 mg/mL in 0.20% Triton X-100. The activity of hexokinase (HX, EC 2.7.1.1) was assayed in the media at a final volume of 0.750 mL. The media contained the following (in mmol/L): 50 Tris/HCl (pH 7.4), 8 MgCl 2, 0.20 NADP, 2 mM glucose, 1U glucose-6-phosphate dehydrogenase, and 0.10–0.30 mg protein. The reaction was initiated by the addition of 0.05 mL 0.1 mM ATP. The enzyme activity was calculated as nmol of reduced NADP based on an absorbance ratio for NADP = 6.22 mol/cm at the wavelength of 340 nm.

The activity of glucose-6-phosphate dehydrogenase (G6P-DH, EC 1.1.1.49) was assayed in the media at a final volume of 0.750 mL. The media contained the following (in mmol/L): 50 Tris/HCl (pH 7.4), 8 MgCl 2, 0.20 NADP, 0.10–0.30 mg protein. The reaction was initiated by the addition of 0.05 mL 0.1 mM glucose-6-phosphate. The enzyme activity was calculated as nmol of oxidized NADH based on an absorbance ratio for NADH = 6.22 mol/cm at the wavelength of 340 nm.

Lactate dehydrogenase (LDH, EC 1.1.1.27) was determined by the direct measurement of NADH oxidation. The incubation media at a final volume of 1.0 mL consisted of the following (in mmol/L): 50 Tris/HCl (pH 7.4), 0.10 NADH, 1 pyruvic acid, and 0.02 mg tested enzyme. The enzyme activity was presented as nmol of oxidized NADH based on an absorbance coefficient for NADH = 6.22 mol/cm at 340 nm.

The activities of aconitase (ACO, EC 4.2.1.3) and isocitrate dehydrogenase (IC-DH, EC 1.1.1.42) were measured by a direct measurement of NADP reduction. The aconitase was assayed in the incubation media with a final volume of 1.0 mL. The media contained the following (in mmol/L): 50 Tris/HCl (pH = 7.4), 2 MgCl_2_, 0.10 NADP, 1 J.M. isocitrate dehydrogenase, and 0.10–0.30 mg of tested protein. The reaction was initiated by the addition of 0.01 mL 20 mM cis-aconitine.

The isocitrate dehydrogenase was assayed in the incubation media at a final volume of 0.6 mL. The media contained the following (in mmol/L): 50 Tris/HCl (pH = 7.4), 2 MgCl_2_, 0.50 NADP, and 0.1 mL tested sample containing 0.1 mg of protein. The reaction was initiated by the addition of 0.01 mL 10 mmol/L isocitrate. The enzyme activity was presented as nmol of reduced NADP based on an absorbance ratio for NADP = 6.22 mol/cm at the wavelength of 340 nm.

Fatty acid synthase (FASN, E.C. 2.3. 1.85) was determined by the direct measurement of NADPH oxidation. The incubation media at a final volume of 0.700 mL consisted of the following (in mmol/L): 80 PBS (pH 7.4), 1 DTT, 0.10 NADPH, 0.10 EDTA, 100 KCl, and 0.10–0.30 mg of tested protein. The reaction was initiated by the addition of 0.01 mL 10 mM malonyl-CoA.

The enzyme activity was presented as nmol of oxidized NADPH based on an absorbance ratio for NADP = 6.22 mol/cm at the wavelength of 340 nm. The total amount of protein was assessed using the Bradford method. The enzymatic activity in each case was calculated considering the amount of protein in the sample and presented as nmol/minute/mg protein. All reagents were purchased from Sigma-Aldrich (Burlington, MA, USA). The absorbance was measured with the UV/Vis UltroSpec3000Pro and UltroSpec3100 Spectrophotometers (Amersham Biosciences, Piscataway, NJ, USA).

### 2.5. mTOR and p38 Phosphorylation

Intracellular Bead-Based Multiplex Assays (MILLIPLEX^®^ 2-Plex Phospho/Total p38 and MILLIPLEX^®^ MAP 2-Plex Phospo/Total mTOR; Merck, Darmstadt, Germany) were used following the manufacturer’s protocols to perform mTOR and p38 MAPK phosphorylation analysis. The assays were analyzed with MAGpix using Luminex XMAP Technology obtained from Merck (Darmstadt, Germany), and the results were obtained with the Milliplex Analyst 5.1 Software. Results are shown as mean fluorescence intensity (MFI) values.

### 2.6. Data Analysis

The flow cytometry data were analyzed using Kaluza Software 2.1 (Beckman Coulter, Brea, CA, USA). Statistical analysis was performed with Statistica 13 (StatSoft, Cracow, Poland) and Prism 9 (GraphPad Software, Boston, MA, USA). The Grubbs test was performed to detect outliers, and only cleaned-up data were used for the statistical analysis. The correlation matrix was performed with GraphPad Prism 9. The Kruskal–Wallis and Mann–Whitney tests were performed to assess the existence of statistically significant differences between T1D and healthy groups or between groups cultured in varying glucose concentrations.

## 3. Results

### 3.1. TIGIT Is Underexpressed in T Cells from the Peripheral Blood of Type 1 Diabetes Patients

**Figure 2:**
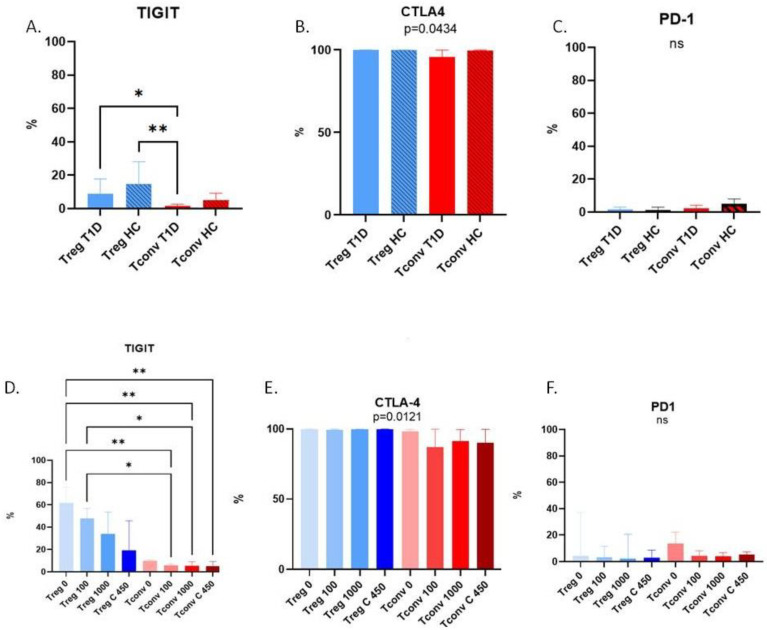
The percentage of regulatory and conventional T cell subpopulations (TIGIT+, CTLA-4+, or PD-1+) in type 1 diabetes (T1D) patients and healthy controls (HCs); (**A**–**C**) comparison of cells’ phenotypes from T1D and HC individuals (stage 1); (**D**–**F**) comparison of cells’ phenotypes from modified HC culture conditions (stage 2). Graphs are shown as medians with interquartile ranges; *p* < 0.05 is considered significant. * *p* < 0.05; ** *p* < 0.01. Kruskal–Wallis with Dunn’s multiple comparisons statistical tests: (**A**) TIGIT, *p* = 0.0029; (**B**) CTLA-4, *p* = 0.0434; (**C**) PD-1, *p* = 0.0883, (**D**) TIGIT, *p* < 0.0001; (**E**) CTLA-4, *p* = 0.0121; (**F**) PD-1, *p* < 0.7588. Treg—T regulatory cells, Tconv—T conventional cells, T1D—type 1 diabetes, HC—healthy control; 0/100/1000/450 correspond to glucose concentrations in cell culture media of 0 mg/dL, 100 mg/dL, 1000 mg/dL in RPMI medium, and 450 mg/dL in XVIVO20 medium.

The comparison of peripheral blood T cell subsets revealed that the percentage of TIGIT+ Tregs differed significantly between T1D patients and healthy controls. The percentages of both TIGIT+ Tregs and TIGIT+ Tconvs was lower in the peripheral blood of T1D patients compared to healthy controls (Figure 2A).

### 3.2. Enzymatic Activity and mTOR and p38 MAPK Phosphorylation in the Peripheral Blood T Cells

There were no differences in the enzymatic activity of Treg and Tconv cells from type 1 diabetes and healthy control patients. The Kruskal–Wallis test with Dunn’s multiple comparisons and the U Mann–Whitney test were performed to assess if the results varied significantly (Supplementary Materials, Figure S1). Furthermore, no differences were identified in the activity of aconitase, lactate dehydrogenase, hexokinase, isocitrate dehydrogenase, or fatty acid synthase cultured in media with different glucose concentrations (0, 100, 1000, and 450 mg/dL) (Supplementary Materials, Figure S1). A significant difference was found in the activity of glucose-6-phosphate dehydrogenase (Supplementary Materials, Figure S2; *p* = 0.0307).

The amount of phosphorylated (*p* = 0.6702) and total mTOR (*p* = 0.1846) did not differ between type 1 diabetes patients (*n* = 8) and healthy controls (*n* = 4), nor did the percentage of phosphorylated mTOR within the total mTOR (*p* = 0.1516) differ between those groups (Supplementary Materials, Figure S3A–E).

Similarly, p38 MAPK showed no difference in total p38 (*p* = 0.2716) or percentage of phospho-p38 within total p38 (*p* = 0.6740) between groups (Supplementary Materials, Figure S3). A difference was found in phosphorylated p38 MAPK (*p* = 0.0325) when comparing Tregs and Tconvs from healthy controls and type 1 diabetes patients (Supplementary Materials, Figure S4). The amounts of p38 (phosphorylated: *p* = 0.3738; total: *p* = 0.7439) and mTOR (phosphorylated: *p* = 0.7654; total: *p* = 0.6535) also did not differ between cell populations cultured under altered glucose availability conditions (Supplementary Materials, Figure S3F–I).

### 3.3. Hyperglycemia Reduces the Percentage of TIGIT+ T Cells and Influences Glucose Metabolism in T Cells

The analysis of associations revealed that TIGIT was the checkpoint inhibitor with the most consistent mode of action when T cells were challenged with a changing concentration of glucose. The percentage of both TIGIT+ Tregs and TIGIT+ Tconvs decreased with increasing concentrations of glucose (Figure 2D).

Interestingly, the decreasing percentage of TIGIT+ Tregs under hyperglycemia in culture was also associated with changes suggesting limited glucose uptake and metabolism in Tregs, such as the decreasing percentage of GLUT3+ Tregs (which allows glucose transport into the cell) and the decreasing activity of hexokinase (which starts glycolysis), isocitrate dehydrogenase (which utilizes pyruvate in the Krebs cycle), and CCR5 (a chemokine that takes part in T cell recruitment and migration). Nevertheless, under these conditions, there was an increasing percentage of mitochondria-active MitoTrackerRed+ Tregs. The decreasing percentage of TIGIT+ Tregs was associated with increasing expression of the CD57 receptor (the marker of immunosenescence) (Table 3) and a decrease in the percentage of CD27-positive Tregs (Table 3 and Supplementary Figure S5).

A similar limitation of glucose metabolism in hyperglycemic conditions was noted in Tconvs. The decreasing percentage of TIGIT+ Tconvs upon hyperglycemia was associated with lower expression of GLUT1 and GLUT3, decreasing activity of G6PDH (pentose phosphate pathway producing glycolysis substrates), and lactate dehydrogenase (metabolizes pyruvate to lactic acid instead of incorporating it into the Krebs cycle). Yet the decreasing percentage of TIGIT+ Tconvs was associated with an increasing percentage of mitochondria-active MitoTrackerRed+ Tconvs and FAS-L+ Tconvs (Table 3).

There was also an association between hyperglycemia and a reduced percentage of CTLA-4+ Tregs (Table 4) but not CTLA-4+ Tconvs. As with TIGIT+ Tregs, the changes in the percentage of CTLA-4+ Tregs suggested reduced glucose metabolism upon activation in hyperglycemic conditions. A decreasing percentage of CTLA-4+ Tregs in hyperglycemic culture was associated with decreased expression of GLUT1, overall reduced phosphorylation of mTOR, and CD39. Similarly to TIGIT+ Tregs and CCR5+ Tregs, the association between CTLA-4+ Tregs, CTLA-4 Tconvs, and CXCR4 was identified, confirming the impairment of the migratory capacity of those cells.

On the other side, a decreasing percentage of CTLA-4+ Tconvs was associated with increasing expression of GLUT-1 and GLUT-3 on these cells and, surprisingly, with a reduced percentage of mitochondria-active MitoTrackerRed+ Tconvs. The reduced percentage of CTLA-4+ Tconvs was also associated with an increased percentage of immunosenescent CD57+ Tconvs.

## 4. Discussion

TIGIT+ Tregs are a highly suppressive subset of T cells, often associated with immune tolerance, tissue protection, and restraint of autoimmunity. In the context of T1D, where autoimmune destruction of pancreatic β-cells is crucial, functional preservation of TIGIT+ Tregs is likely critical for preventing or slowing disease progression.

As the levels of TIGIT+ Tregs, CTLA-4+ Tregs, and TIGIT+ Tconvs decreased with increasing glucose concentrations, we hypothesized that the functionality of these T cell subpopulations could be significantly influenced by the metabolic state of the cell.

Higher ambient glucose reduces TIGIT+ Tregs. In our study, we found that this state is also characterized by decreased glucose uptake and metabolism in Tregs (positive association of TIGIT+ Tregs with GLUT3+ Tregs, hexokinase, and isocitrate dehydrogenase activity in Tregs). Moreover, hyperglycemia, along with low TIGIT+ Tregs levels, results in the induction of immune senescence (negative association between TIGIT+ Tregs and CD57+ Tregs).

Joller et al.’s research shows that TIGIT+ Tregs can share some features with proinflammatory T cells, highlighting the role of chemokines in directing them to the site of inflammation [15]. In our study, we found positive associations between CCR5+ Tregs and TIGIT+ Tregs, as well as between CXCR4+ Tregs and CTLA-4 Tregs. This confirms the impairment of the migratory capacity of Tregs, making them unable to properly suppress proinflammatory T cells in the pancreas. Additionally, CCR5+ cells were found to play a critical role in autoimmune disorders, not only in cell migration but also in affecting the suppressive properties of Tregs from T1D patients [16]. Our studies seem to confirm this thesis, as we found a positive association between highly proinflammatory TIGIT+ Tregs and CCR5+ Tregs.

The first screening resulted in a negative association between TIGIT+ and CD27+ Tregs. Further analysis showed that this result interfered with the results obtained from the control group (Supplementary Figure S5). This graph shows that the proportion of CD27+ Tregs decreases with the increasing concentration of glucose in the medium, which seems to confirm the results of our and other studies showing that CD27 expression is a marker of the suppressive population [17].

The reduction in CTLA-4+ Tregs in hyperglycemia coincided with a metabolic shift marked by decreased GLUT1 expression and overall downregulation of mTOR phosphorylation. These findings indicate suppressed glucose metabolism accompanying the state of weakened suppressive properties of Tregs, represented by low CTLA-4 expression.

Decreased CTLA-4+ Tregs in hyperglycemic conditions may also represent abnormal Treg cell function in autoimmune diabetes [18]. Similarly to TIGIT+ Tregs, the lowered percentages of CTLA-4+ Tregs were associated with decreased glucose uptake. Furthermore, the amount of total and phosphorylated mTOR, which is directly correlated with glucose metabolism [19,20], and CD39+ Tregs, representing the metabolism of ATP into immunomodulatory adenosine [21], also dropped along with CTLA-4-positive Tregs in hyperglycemia.

On the other hand, extending the analysis to conventional T cells may reveal the elements of the disease pathomechanism. TIGIT expression on Tconvs (non-regulatory CD4+ cells) typically indicates the state of cell exhaustion in chronic inflammation [22,23]. Following repeated activation, conventional T cells within the population may undergo apoptosis through Fas–FasL interactions, whereby FasL-expressing T cells induce cell death in neighboring Fas-expressing T cells. This process, known as activation-induced cell death (AICD), helps maintain immune homeostasis and prevent autoimmunity by eliminating overactive or self-reactive T cells [24,25,26]. In a high-glucose environment, the TIGIT+ Tconvs drop. The negative association between TIGIT+ Tconvs and FAS-L+ Tconvs may suggest that hyperglycemia can inhibit cell apoptosis, leading to the accumulation of unchecked effector T cells and worsening β-cell destruction in diabetic conditions.

Despite reduced glucose uptake, suppressed glycolysis, and TCA cycle activity, an increase in mitochondria-active T cells indicates a shift towards alternative metabolic pathways or a compensatory response to maintain cellular energy homeostasis. This could involve enhanced fatty acid oxidation or other mitochondrial processes that are less reliant on glucose metabolism. Notably, this metabolic profile is characteristic of memory or resting T cells, suggesting that these cells may be transitioning toward a long-lived, quiescent state supported by lipid metabolism [19]. The percentages of CTLA-4+ T conventional cells in hyperglycemic conditions were associated GLUT1-positive and GLUT3-positive Tconvs and decreased mitochondrial activity, confirming the inhibitory effect of CTLA-4 stimulation on glycolysis in Tconvs [19].

Collectively, these findings underscore that hyperglycemia differentially impacts metabolic programming in Tregs and Tconvs, leading to altered expression of key immune checkpoints, such as TIGIT and CTLA-4, as well as other molecules that support Tregs’ functionality, such as CD39, or the expression of chemokines (CCR5 and CXCR4). The observed metabolic plasticity, particularly in Tregs, may have profound implications for their immunosuppressive function in hyperglycemic environments such as those seen in diabetes.

The functional meaning of TIGIT expression on T cells is context-dependent and differs between Tconvs and Tregs. TIGIT expression on Tconvs has been increasingly linked to sustained inhibitory signaling, reduced effector function, and features consistent with T cell exhaustion rather than transient activation [27]. In contrast, TIGIT expression on Tregs has been associated with enhanced suppressive capacity, lineage stability, and effective control of inflammatory T cell responses. Thus, changes in TIGIT expression may result in divergent functional consequences for Tregs and Tconvs, reflecting their distinct roles within the immune system.

In the context of type 1 diabetes, persistent hyperglycemia may directly impair the most functionally suppressive TIGIT+ Treg subset, which plays a role in maintaining peripheral tolerance. Simultaneously, the reduction in the percentage of exhausted TIGIT+ Tconvs suggests the inability of the system to maintain immune homeostasis, tipping the balance toward autoimmunity. Additionally, it confirms that well-controlled diabetes, by keeping glucose values within normal limits, has a beneficial impact by preserving regulatory T cell function and mitigating inflammatory T cell responses.

Our research, along with that of others in the field [10,27,28,29,30], demonstrates that TIGIT, similar to PD-1 and CTLA-4, could be another critically important molecule in effectively managing autoimmune diseases. These findings may provide a rationale for therapeutic strategies aimed at enhancing in vivo TIGIT expression on T cells to restore immune homeostasis and ameliorate autoimmunity [6,31].

Further research is warranted to delineate the mechanistic underpinnings of these metabolic shifts and to explore their relevance in vivo.

### 4.1. Conclusions

Hyperglycemia profoundly alters T cell metabolism and immune checkpoint expression, leading to impaired regulatory T cell function in T1D. Elevated glucose levels are associated with a reduction in highly suppressive TIGIT+ and CTLA-4+ Tregs, accompanied by decreased glucose uptake. In parallel, a decrease in TIGIT+ Tconvs under hyperglycemic conditions may limit activation-induced cell death, promoting the accumulation of unchecked effector T cells driving autoimmune processes.

These findings highlight TIGIT as a key immunometabolic regulator linking glucose metabolism to immune tolerance and suggest that maintaining normoglycemia or targeting TIGIT signaling may help restore immune balance in autoimmune diabetes.

### 4.2. Study Limitations

The control group differed significantly in age compared to the study group, as type 1 diabetes typically manifests in childhood and adolescence, whereas blood donation is largely restricted to adults. These differences were inherent to the recruitment process, as the availability of healthy donors matching the study group’s age range was limited.

Age-related changes are more pronounced in naive and memory T cell compartments, whereas the relative frequency of regulatory T cells has been reported to remain stable or to increase modestly with age. Therefore, we believe that the observed alterations are not age-dependent.

Another limitation of this study is the omission of CD73 in the phenotyping panel of cells, with only CD39 being included [32]. CD39 is responsible for the conversion of ATP and ADP into AMP; the subsequent step of converting AMP into adenosine is covered by CD73. Therefore, the absence of CD73 data restricted our ability to fully characterize the pathway’s functionality and the potential immunosuppressive phenotype of the cells. Future studies should incorporate both CD39 and CD73 in the phenotyping panel to enable a more comprehensive analysis of the purinergic signaling pathway and its impact on cellular function and immune regulation. Further research involving a larger study group is required to give the presented results greater statistical power.

## Figures and Tables

**Table 1:** Patients’ characteristics, **** *p* < 0.0001.

Parameter	Type 1 Diabetes	Healthy Controls	Graph
Age [median; min–max] Mann–Whitney test	12; 7–15	27; 19–43	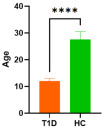 **** *p* < 0.0001
Sex [%] (male/female)	81/19	69/31	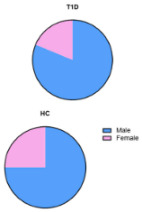

**Table 2:** The flow cytometry antigen panel.

	Control	Maturation and Differentation	Suppression	MitoTracker
Antigen (clone) fluorochrome	CD4 (RPA-T4) BUV 395	CD4 (RPA-T4) BUV 395	CD4 (RPA-T4) BUV 395	CD4 (RPA-T4) BUV 395
	CD3 (UCHT1) Pacific Orange	CD3 (UCHT1) Pacific Orange	CD3 (UCHT1) Pacific Orange	CD3 (UCHT1) Pacific Orange
		CD45RA (HI100) SuperBright 600	CD45RA (HI100) SuperBright 600	
		CD62L (DREG-56) APC-eFluor 780	CD62L (DREG-56) APC-eFluor 780	
		CD27 (O323) SuperBright 645	CCR5 (3A9) BV 650	
		CD279 (J105) SuperBright 702	CD39 (TU66) BV 711	
		CD178 (NOK-1) PE	CD152 (14D3) PE	
		CD28 (CD28.2) PE-Cy7	CD184 (12G5) PE-Cy7	
		CD57 (TB01) eFluor 450	TIGIT (MBSA43) eFluor 450	
		GLUT 1 (202915) APC	GLUT 1 (202915) APC	
		GLUT 3 (202017) Texas Red	GLUT 3 (202017) Texas Red	
		FoxP3 (PCH101) Per CP-Cy5	FoxP3 (PCH101) Per CP-Cy5	
		Helios (22F6) Alexa 700	Helios (22F6) Alexa 700	
		MitoTracker Green	MitoTracker Green	MitoTracker Green
				MitoTracker Red

**Table 3:** Associations between TIGIT+ Tregs or Tconvs and other parameters. Spearman’s r test. Results from stage 2 of analysis.

Parameter	Association	Spearman r	*p*
Treg			
CCR5	Positive	0.587	0.0051
GLUT 3	Positive	0.5	0.0508
Isocitrate dehydrogenase	Positive	0.3315	0.0912
Hexokinase	Positive	0.5709	0.0055
CD27	Negative	−0.509	0.007
Glucose concentration	Negative	−0.4315	0.0246
MitoTracker Red CXMRos	Negative	−0.4283	0.099
CD57	Negative	−0.4345	0.0265
Tconv			
GLUT1	Positive	0.6834	0.0062
GLUT3	Positive	0.7069	0.0029
Glucose-6-phosphate dehydrogenase	Positive	0.4047	0.0448
Lactate dehydrogenase	Positive	0.4617	0.0153
Glucose concentration	Negative	−0.3583	0.0665
MitoTracker Red CXMRos	Negative	−0.5048	0.0481
FAS-L	Negative	−0.63	0.0238

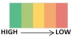
.

**Table 4:** Associations between CTLA-4 Tregs or Tconvs and other parameters. Spearman’s r test. Results from stage 2 of analysis.

Parameter	Association	Spearman r	*p*
Treg			
GLUT1	Positive	0.5559	0.0337
tmTOR	Positive	0.5245	0.0839
pmTOR	Positive	0.6364	0.0299
CD39	Positive	0.5704	0.0056
CXCR4	Positive	0.4917	0.0467
pp38	Negative	−0.578	0.067
Glucose concentration	Negative	−0.3864	0.057
Tconv			
CXCR4	Positive	0.4535	0.0512
MitoTracker Red CXMRos	Positive	0.7653	0.0008
FAS-L	Positive	0.638	0.052
GLUT1	Negative	−0.4794	0.0722
GLUT3	Negative	−0.4352	0.0927
CD57	Negative	−0.4516	0.0399

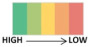
.

## Data Availability

The data that support the findings of this study are available from the corresponding author upon reasonable request.

## Supplementary Materials

The following supporting information can be downloaded at https://www.mdpi.com/article/10.3390/cells15020195/s1: Figure S1: The activity of enzymes in regulatory and conventional T cells. Figure S2: The activity of glucose-6-phosphate dehydrogenase [nmol/min/mg of protein] in T regulatory and T conventional cells cultured in a medium with 0/100/1000/450 mg/dL of glucose. Figure S3: MFI of phosphorylated and total p38MAPK and mTOR kinases from regulatory and conventional T cells. Figure S4: MFI of phosphorylated p38 MAP kinase in T regulatory and T conventional cells from healthy controls and type 1 diabetic patients. Figure S5: The percentage of CD27 (with or without the control group) expressing Tregs in cell cultures with different glucose concentration Figure S6: Gating strategy.

## Abbreviations

The following abbreviations are used in this manuscript:

ACO	Aconitase
CD	Cluster of differentiation
CTLA-4	Cytotoxic T lymphocyte antigen 4
DTT	Dithiothreitol
EDTA	Ethylenediaminetetraacetic acid
FAS	Fatty acid synthase
FoxP3	Transcriptional factor forkhead box P3
G6P	Glucose-6-phosphate dehydrogenase
GLUT	Glucose transporter
GMP	Good manufacturing practice
HC	Healthy control
HX	Hexokinase
IDH	Isocitric dehydrogenase
IL	Interleukin
LDH	Lactate dehydrogenase
MAPK	Mitogen-activated protein kinase
MFI	Mean fluorescence activity
mTOR	Mechanistic target of rapamycin
NAD	Nicotinamide adenine dinucleotide
NADH	Reduced form of nicotinamide adenine dinucleotide
NADP	Nicotinamide adenine dinucleotide phosphate
NADPH	Reduced form of nicotinamide adenine dinucleotide phosphate
PBMC	Peripheral mononuclear blood cell
PBS	Phosphate-buffered saline
PD-1	Programmed cell death receptor 1
PDL1	Ligand for protein cell death protein 1
T1D	Type 1 diabetes
Tconv	T conventional cells/T effector cells
TCR	T cell receptor
TIGIT	T cell immunoreceptor with Ig and ITIM domains
Treg	T regulatory cell
