# Supplementary material for: Reduced TIGIT Expression on T Cells Links Hyperglycemia to Immune Dysregulation in Type 1 Diabetes
Source: Cells. 2026 Jan 20;15(2):195. doi: 10.3390/cells15020195 (PMC12840437; doi:10.3390/cells15020195)
Supplement: Supplementary file 1 [file cells-15-00195-s001.zip › cells-4084278-supplementary.pdf]

Supplementary Materials

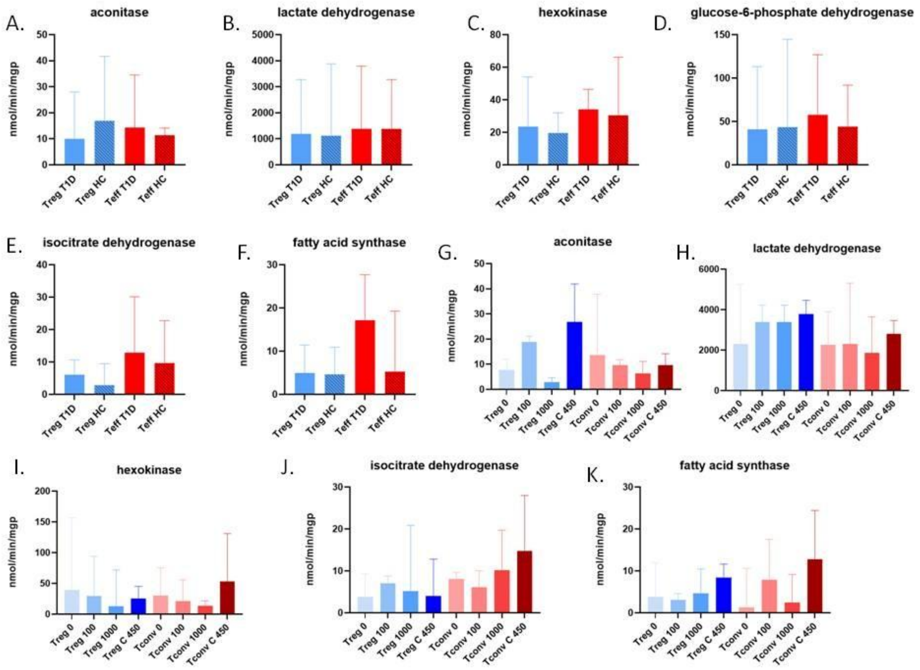

Supplementary Figure S1. The activity of enzymes in regulatory and conventional T cells. Graphs are shown as medians with interquartile range; $p<0.05$ is considered significant. Kruskal- Wallis with Dunn's multiple comparisons statistical tests: A: akonitase p=0.9024; B: lactate dehydrogenase p=0.8596; C: hexokinase p=0.3152, D: glucose-6-phosphate dehydrogenase p=0.9110, E: isocitrate dehydrogenase p=0.0799, F: fatty acid synthase p=0.2365; G: akonitase p=0.1260; H: lactate dehydrogenase p=0.7472; I: hexokinase p=0.6461; J: isocitrate dehydrogenase p=0.3504; K: fatty acid synthase p=0.5999. Treg- T regulatory cells, Tconv- T conventional cells, T1D- type 1 diabetes, HC- healthy control, 0/100/1000/450 corresponds to glucose concentration in cell culture medium 0mg/dl; 100mg/dl;1000mg/dl in RPMI medium; 450mg/dl in XVIVO20 medium.

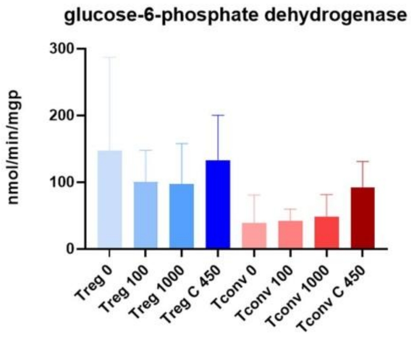

Supplementary Figure S2. The activity of glucose-6-phosphate dehydrogenase [nmol/min/mg of protein] in T regulatory and T conventional cells cultured in a medium with 0/100/1000/450 mg/dl of glucose. Graphs shown as median with interquartile range, $p<0.05$ is considered significant. The Kruskal-Wallis with Dunn's multiple comparison statistical test p=0.0307. Further analysis has shown the difference between T regulatory cells 450mg/dl of glucose and T conventional cells 0mg/dl of glucose (Mann Whitney test 0.0173).

Treg- T regulatory cells, Tconv- T conventional cells, 0/100/1000/450 corresponds to glucose concentration in cell culture medium 0mg/dl; 100mg/dl;1000mg/dl in RPMI medium; 450mg/dl in XVIVO20 medium.

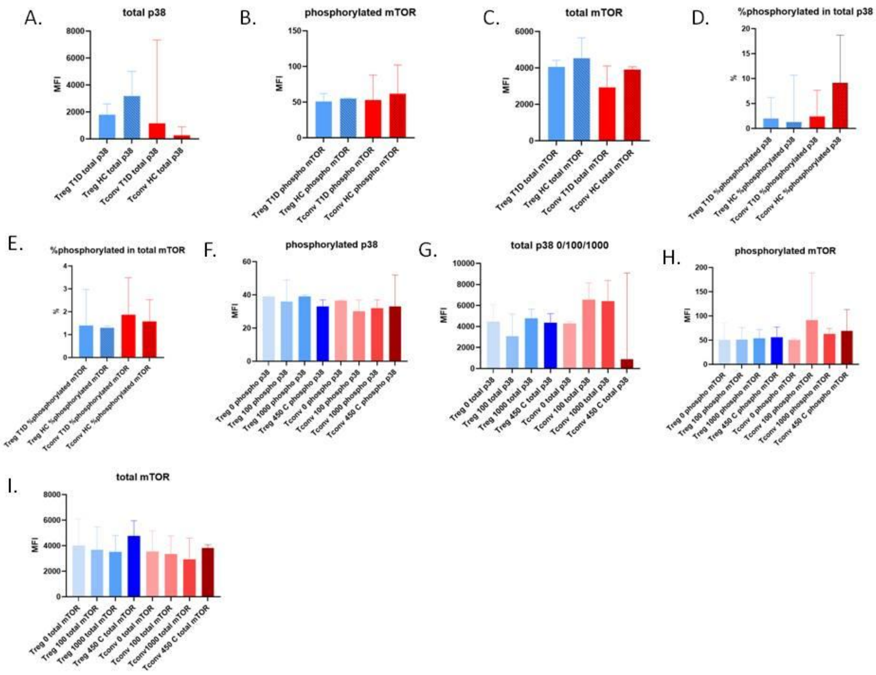

Supplementary Figure S3. MFI of phosphorylated and total p38MAPK and mTOR kinases from regulatory and conventional T cells. Graphs shown as median with interquartile range, p<0.05 is considered significant. Kruskal- Wallis with Dunn's multiple comparisons statistical tests: A: akonitase p=0.9024; B: lactate dehydrogenase p=0.8596; C: hexokinase p=0.3152, D: glucose-6-phosphate dehydrogenase p=0.9110, E: isocitrate dehydrogenase p=0.0799, F: fatty acid synthase p=0.2365; G: akonitase p=0.1260; H: lactate dehydrogenase p=0.7472; I: hexokinase p=0.6461; J: isocitrate dehydrogenase p=0.3504; K: fatty acid synthase p=0.5999. Treg- T regulatory cells, Tconv- T conventional cells, T1D- type 1 diabetes, HC- healthy control, 0/100/1000/450 corresponds to glucose concentration in cell culture medium 0mg/dl; 100mg/dl;1000mg/dl in RPMI medium; 450mg/dl in XVIVO20 medium.

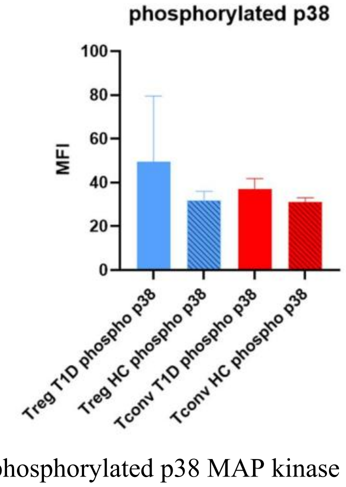

Supplementary Figure S4. MFI of phosphorylated p38 MAP kinase in T regulatory and T conventional cells from healthy controls and type 1 diabetic patients. Graphs shown as median with interquartile range, p<0.05 is considered significant, *p<0.05. The Kruskal-Wallis with Dunn's multiple comparison statistical test p=0.0325. Treg- T regulatory cells, Tconv- T conventional cells, T1D-type 1 diabetes, HC- healthy control.

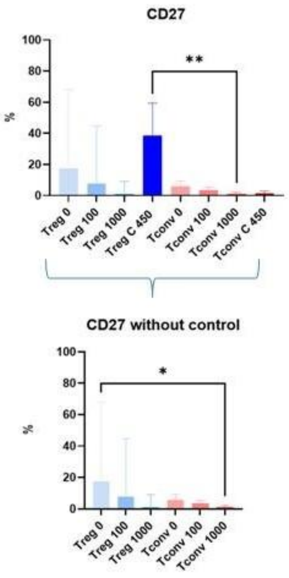

Supplementary Figure S5. The percentage of CD27 (with or without the control group) expressing Tregs in cell cultures with different glucose concentrations. Treg- T regulatory cells, Tconv- T conventional cells, C- control medium; 0/100/1000/450 corresponds to glucose concentration in cell culture medium 0mg/dl; 100mg/dl;1000mg/dl in RPMI medium; 450mg/dl in XVIVO20 medium.

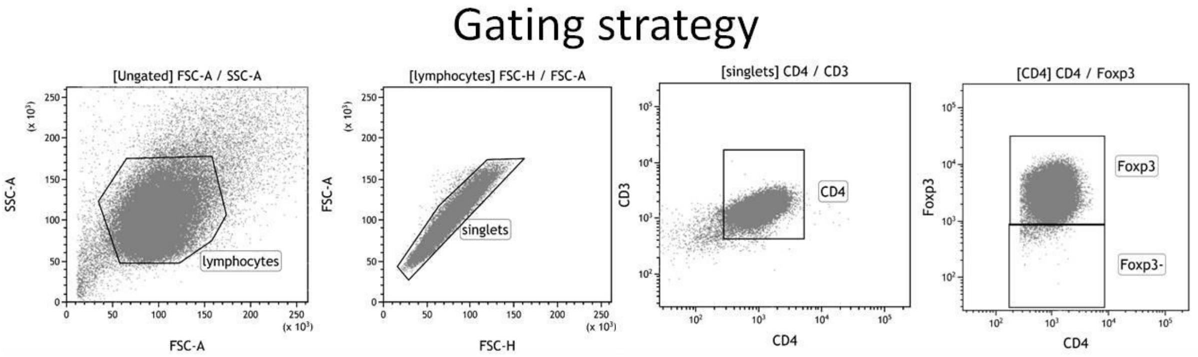

Supplementary Figure S6. Gating strategy.

In the study as a minimum backbone marker strategy was applied, where a fixed set of well-established bacbone markers (CD3 and CD4) was used to define major lineages. Additional markers were interpreted within these clearly defined populations. This approach reduces the reliance on isotype controls and is commonly applied in multicolor flow cytometry analyses. Gates for minor populations were defined based on backone marker expression, internal negative populations, and established biological patterns reported in the literature.
